# A Multidisciplinary Occupational Medicine-Based Intervention Protocol for Conflict Prevention and Crisis Management in High-Stress Professional Environments

**DOI:** 10.3390/brainsci15090958

**Published:** 2025-09-02

**Authors:** Martina Corsi, Dorotea Stefanini, Isabella Biagioni, Chiara Bertini, Matteo Accardo, Mirko Bottari, Claudia Antunes, Laura Lazzarini, Ilaria Pertici, Chiara Ciarfella, Giovanni Tritto, Salvio Perretta, Poupak Fallahi, Rudy Foddis

**Affiliations:** 1 Occupational Health Department, Azienda Ospedaliero-Universitaria Pisana, Via Paradisa 2, 56124 Pisa, Italy; salvio.perretta@gmail.com (S.P.); poupak.fallahi@unipi.it (P.F.); rudy.foddis@unipi.it (R.F.); 2 Department of Translational Research and New Technologies in Medicine and Surgery, University of Pisa, 56126 Pisa, Italy; dorotea.stefanini@gmail.com (D.S.); isabellabiagioni@gmail.com (I.B.); bertinichiara94@gmail.com (C.B.); matteo.accardo95@gmail.com (M.A.); mirko.bottari@gmail.com (M.B.); cl_dantunes@hotmail.com (C.A.); l.lazzarini6@studenti.unipi.it (L.L.); ilariapertici1996@gmail.com (I.P.); chiara-c-92@hotmail.it (C.C.); giovanni_tritto@hotmail.it (G.T.)

**Keywords:** workplace violence, conflict management, crisis intervention, psychosocial risks, occupational health, professional training, role-playing, de-escalation

## Abstract

**Background/Objectives:** Workplace conflict and aggression pose significant psychosocial risks across diverse professional sectors. This protocol outlines a novel, university-based educational intervention. Developed by a multidisciplinary team from the University Hospital of Pisa, Italy, including occupational physicians and a psychiatrist specializing in work and organizational psychology, its primary purpose is to enhance conflict prevention and crisis management skills. While initially developed and tested within the veterinary sector due to its identified vulnerabilities, the intervention is inherently generalizable to any high-stress professional environment characterized by intense client, customer, or public interactions. **Methods:** The intervention integrates didactic instruction with active, immersive learning through tailored role-playing scenarios simulating real-world challenging encounters. This study protocol details the structured methodology for evaluating the immediate effectiveness of this training. We are using a specifically developed efficacy scale to assess outcomes. **Results:** The results demonstrate a significant improvement in all assessed skills from the pre-training to the post-training evaluation. For every item on the scale, the median scores increased, indicating a positive shift in overall group performance. The *p*-value for each item was <0.001, confirming that the observed improvements were statistically significant. These results demonstrate enhanced conflict resolution skills, improved communication, and an increased sense of self-efficacy among participants. **Conclusions**: This protocol offers a comprehensive and generalizable approach to addressing workplace psychosocial risks through an innovative educational intervention. A key future goal involves advancing this training methodology by integrating virtual reality (VR) environments with AI-driven avatars for role-playing, aiming to achieve a more realistic and impactful learning experience and sustained behavioral change.

## 1. Introduction

Workplace violence and aggression constitute a pervasive and escalating global concern, recognized as a significant psychosocial risk that impacts employee well-being, organizational productivity, and the overall work environment [1]. International bodies, such as the International Labour Organization (ILO), have underscored the urgency of addressing these issues, culminating in the adoption of Convention No. 190 in 2019, which explicitly calls for comprehensive measures to prevent and eliminate violence and harassment in the world of work [2]. This convention highlights that such risks are not confined to specific industries but are prevalent across diverse professional settings, particularly those involving substantial public interaction, emotional labor, or high-stakes decision-making. The definition of workplace violence is evolving, moving beyond solely physical assaults to encompass a broader spectrum of behaviors, including threatening conduct and verbal abuse. This expansion reflects a more comprehensive understanding of the harm that can occur in professional settings [3,4].

The World Health Organization (WHO) broadly defines violence as “the intentional use of physical force or power, threatened or actual, against oneself, another person, or against a group or community, that causes or has a high probability of causing injury, death, psychological harm, developmental maldevelopment or deprivation” [5]. While this provides a foundational understanding, implementing prevention strategies necessitates a nuanced grasp of the specific dynamics and vulnerabilities across different professions.

Further broadening this understanding, the National Institute for Occupational Safety and Health (NIOSH) defines workplace violence as “any physical assault, threatening behavior, or verbal abuse that occurs on the job” [6]. More comprehensively, the International Labor Organization (ILO) defines it as “a range of unacceptable behaviors and practices, or threats thereof, whether a single incident or repeated, that aim at, or are likely to result in physical, psychological, sexual or economic harm, and includes gender-based violence and harassment” [2]. This ILO definition is crucial as it formally recognizes the multifaceted nature of workplace violence, explicitly including gender-based violence and harassment.

The workplace violence prevention strategy encompasses some sensitive issues such as psychosocial risks, which include conflicts, stress, and harassment, as well as gender issues. Psychosocial risks, stemming from inadequate work organization and unfavorable social contexts, can lead to psychological, psychiatric, and physical harm [7,8]. Recent literature highlights that violence in the workplace is deeply rooted in gendered socio-economic, cultural, and institutional factors [2,8]. Studies consistently show that women are more likely to experience non-physical violence, such as verbal abuse, sexual harassment, and bullying, while men may experience more physical violence. Gendered power relations disproportionately affect women, inherently increasing their vulnerability to specific forms of workplace violence due to these systemic imbalances [9,10].

The escalation of conflict into violence is also a recognized pathway. Unresolved workplace conflicts, often driven by personality clashes, stress, and heavy workloads, can escalate if not managed effectively [11]. This emphasizes the need for comprehensive strategies that address not only overt acts of aggression but also underlying conflicts and systemic issues, including those related to gender inequality, to foster safer and more respectful work environments [11,12].

Healthcare settings, for instance, are particularly prone to incidents of aggression, as evidenced by recent scoping reviews that identify healthcare workers as a highly vulnerable population [13]. These reviews emphasize the need for evidence-based de-escalation training programs as a primary preventative measure. Our group has previously highlighted the importance of such interventions, particularly in healthcare, acknowledging that effective training must be grounded in recognized best practices to address the nuances of aggressive behavior and foster resilience [11]. Similarly, the veterinary sector, despite being less frequently studied than human healthcare, presents a unique set of challenges. Veterinary professionals routinely manage emotionally charged situations involving pet owners who may be stressed, grieving, or financially burdened, making them particularly susceptible to client dissatisfaction escalating into conflict or even aggression [14,15]. Unmanaged conflict in any professional context can lead to severe consequences, including increased employee stress, burnout, reduced job satisfaction, high staff turnover, damage to an organization’s reputation, and ultimately, decreased quality of service. In extreme cases, it can result in physical injury or psychological trauma [16].

Given these pervasive challenges, there is a pressing need for structured, adaptable, and evidence-informed educational interventions that empower professionals with practical skills to prevent conflicts and effectively manage crises. Current literature supports the efficacy of training programs that combine theoretical knowledge with practical skill-building, particularly through simulated scenarios [11,17]. While traditional role-playing offers significant benefits, future advancements aim to enhance realism and effectiveness through immersive technologies [11,18,19].

This protocol describes an intervention developed by a multidisciplinary team from the Unit of Occupational Medicine at the University Hospital of Pisa, Italy. While its initial application and refinement were conducted within the veterinary context, the core principles and methodologies are designed for broad generalizability, aiming to establish a replicable framework for psychosocial risk management from an occupational health perspective across various high-stress professional environments.

The primary objectives of this protocol are as follows:

1. To detail the design and theoretical underpinnings of a comprehensive educational intervention program focused on conflict prevention and crisis management.
2. To outline a robust methodology for assessing the immediate impact of this training on participants’ abilities to handle challenging interactions, utilizing immersive role-playing scenarios and a specifically developed efficacy scale.
3. To present this protocol as an adaptable model for organizations and occupational health services seeking to enhance professional resilience and mitigate psychosocial risks within their workforce across diverse sectors, also setting the stage for future technological advancements in training methodologies.

## 2. Materials and Methods

### 2.1. Study Design and Setting

A detailed schedule of the project and intervention’s phases are described in Table 1.

This study outlines an educational intervention protocol structured as a three-phase program designed to enhance skills in conflict prevention and crisis management. The program was developed by a multidisciplinary team comprising university occupational physicians, resident physicians in training, and a psychiatrist who is also a psychotherapist with specific training in work and organizational psychology from the Unit of Occupational Medicine, University Hospital of Pisa, Italy. The conceptualization of this protocol is rooted in a comprehensive understanding of psychosocial risks and best practices in workplace aggression management, informed by recent systematic reviews [11]. While the initial implementation targets veterinary professionals, the protocol’s design explicitly ensures its modularity and adaptability for implementation in other high-stress professional settings where interactions with clients/public can escalate into conflict.

### 2.2. Participants

The target audience for this protocol encompasses professionals working in environments characterized by frequent, often emotionally charged, interactions with clients, customers, or the public. This includes, but is not limited to, healthcare workers (human and veterinary), customer service representatives, educators, social workers, and administrative staff who routinely face situations with a high potential for conflict or aggression. For any specific implementation, participants will be selected based on their role and exposure to challenging interactions. Ethical considerations, including obtaining informed consent and ensuring data privacy, are paramount for any practical application of this protocol. The intervention program consists of two main phases, designed to move participants from baseline assessment through theoretical learning to skill consolidation and reassessment. Each phase is carefully structured to maximize learning and retention.

The heterogeneous composition by role and age was intentionally designed to ensure that the protocol’s effectiveness could be tested across a broad cross-section of professionals, confirming its applicability to various high-stress work contexts (see Table 2).

### 2.3. Intervention Program: Length, Structure and Content

The overall duration of the program was kept brief for a specific reason: to facilitate long-term skill transfer, the short, focused sessions were structured according to the principle of spaced repetition, thus allowing participants the necessary time to apply new knowledge between sessions [20].

Phase 1: Initial Competency Assessment (Role-Playing Session 1).

Description: This phase involves an initial, brief (approximately 5 min per participant) individual role-playing session. Participants are presented with simulated conflict scenarios typical of their professional environment. For instance, in the veterinary context, this could involve an agitated pet owner, while in a customer service setting, it might be a highly dissatisfied client.

Methodology: No prior training or guidance was provided before this session. Participants were expected to rely solely on their existing professional sensitivity, experience, and knowledge to manage the simulated situation. The scenarios were tailored to the specific role of the participant (e.g., healthcare, technical, or administrative personnel).

Observation and Evaluation: During the session, a team of external raters, positioned discreetly to avoid influencing the interaction, observed the participant’s performance. These raters completed a specifically developed “efficacy scale” to objectively measure the participant’s baseline capacity for conflict management and de-escalation. Crucially, the raters did not intervene during the role-play. This initial assessment provides a non-judgmental baseline for group performance, serving as a point of comparison for the post-training assessment. To ensure an authentic assessment, direct comparison or preparation among participants before this session was discouraged.

Phase 2: Training and Skill Consolidation.

Theoretical Meetings: This phase comprised a total of two hours of structured theoretical instruction. The content is universally applicable to various professional contexts and is designed to provide a comprehensive understanding of conflict dynamics and effective management of de-escalation strategies. Key topics include:

- Understanding Workplace Violence and Psychosocial Risks: Definitions of violence (drawing from WHO and NIOSH) and a comprehensive overview of psychosocial risks in the workplace, including their sources and impacts. This section emphasizes the proactive identification and mitigation of these risks from an occupational health perspective.
- Dynamics and Phases of Aggression: A detailed exploration of the typical phases of aggressive behavior—prodromal (pre-aggression), acute (active aggression), and post-critical depression (de-escalated, reflective state). For each phase, specific, universally applicable de-escalation and management strategies are discussed.

Prodromal Phase: This focuses on recognizing early warning signs (e.g., changes in tone, body language, agitation). Management strategy emphasizes maintaining a calm, professional demeanor and assessing the situation for potential de-escalation opportunities.

Acute Phase: Emphasis on safety as the paramount concern. Strategies include avoiding direct confrontation, utilizing “synthetic options” such as physical containment (if safe and appropriate), strategic withdrawal and self-protection. The critical directive is “Do not try to calm down the aggressive subject” in a confrontational manner, but rather to focus on reducing consequences and ensuring safety. Intervention should not be conducted on the premise of a rational response during acute aggression.

Post-Critical Depression Phase: Characterized by feelings of guilt, shame, or remorse in the aggressive individual. This phase is conducive to psychological interventions aimed at processing the event. The strategy involves discussing the event to reduce the impact of unpleasant emotions and establish a basis for future alliance.

Effective Communication for De-escalation: Fundamental principles of communication crucial for managing difficult interactions are taught. These include the following:

- “Ascolta per validare, non per giudicare” (Listen to validate, not to judge)—promoting empathy and understanding.
- “Mantieni la calma per capire, non per reagire” (Stay calm to understand, not to react)—emphasizing emotional regulation and self-awareness.
- “Osserva le emozioni, non solo le parole” (Observe emotions, not just words)—highlighting the importance of non-verbal cues and emotional intelligence.
- “Rispetta con il corpo” (Respect with your body)—focusing on non-threatening body language and maintaining appropriate personal space to convey respect and minimize escalation.
- “Esponi soluzioni, non problemi” (Propose solutions, not problems)—fostering a constructive and problem-solving approach to conflict.

Phase 3: Final Competency Assessment (Role-Playing Session 2).

After the theoretical training, participants engaged in a second individual role-playing session (approximately 3–5 min). These scenarios were new but of similar complexity to the initial assessment, designed to assess the practical application of the newly acquired knowledge and skills and gauge the impact of the training. The second role-playing session was designed as both an evaluative and a training tool. Following the simulation, participants received personalized feedback that went beyond simple assessment, focusing on both positive performance and areas for improvement. This feedback includes a clear rationale for skill enhancement. A dedicated self-assessment period further facilitates critical reflection and the active consolidation of learning.

The protocol’s modular method is highly adaptable to various professional settings because the fundamental dynamics of human conflict are consistent across different environments. The program is effectively adapted by tailoring role-playing scenarios to the specific challenges of each field.

### 2.4. Evaluation Methods

Performance Assessment: The “efficacy scale,” a standardized tool developed by the multidisciplinary team, was completed by external raters during both the initial and final role-playing sessions. While the application of this scale is integral to evaluating the training program, the validation of its integrated Italian version (Italian Modified Version of the De-Escalation Aggressive Behavior Scale, IMDABS) is the subject of a separate, parallel research project conducted by the Unit of Occupational Medicine, University Hospital of Pisa. This distinct validation study ensures the psychometric robustness of the scale for the Italian context. In this training program, the efficacy scale quantifies observable behaviors and the overall effectiveness of the participant in managing and de-escalating the simulated conflict. The scale focuses on criteria reflecting the theoretical principles taught, such as communication clarity, emotional regulation, de-escalation techniques, and maintaining a professional demeanor. The inter-rater reliability among the three raters was measured using the Intraclass Correlation Coefficient (ICC). The ICC values for each item were all significant with *p* < 0.0001. The internal consistency of the scale, measured with Cronbach’s alpha, was 0.951.

- Feedback and Improvement: Crucially, the results of the performance analysis are aggregated and shared as group-level feedback, not individual scores. This approach is explicitly designed to foster a collaborative and supportive learning environment, encouraging collective reflection and identifying common strengths and areas for collective improvement. This method promotes professional growth without creating competitive pressure among participants, allowing the group to understand the overall impact of the training program and informing its continuous refinement.

#### The IMDABS Scale

The IMDABS (Italian Modified De-escalating Aggressive Behaviors Scale) is an evaluation tool designed to measure de-escalation skills, with responses on a Likert scale from 1 (Strongly disagree) to 5 (Strongly agree). The scale focuses on eight behavioral areas:

- Validating reasons: Acknowledging the concerns of the colleague/client/patient.
- Reducing fear: Actively listening with genuine empathy and offering positive future perspectives.
- Inquiring about doubts: Making an effort to understand the concerns and the root of the problem.
- Providing guidance: Suggesting different ways to help with current concerns and recommending preventative measures.
- Developing possible agreements: Taking responsibility for the management of the case and agreeing on an action plan.
- Maintaining calm: Keeping a calm and steady voice and pace.
- Implementing cautious attitudes/behaviors: Maintaining a moderate distance to ensure safety without appearing guarded or fearful.
- Using appropriate body language: Having adequate eye contact, spontaneous facial expressions, and a relaxed posture

### 2.5. Ethical Considerations

This protocol adheres to the principles of the Declaration of Helsinki. Formal ethical approval has been obtained from the University Bioethics Committee of the University Hospital of Pisa, with resolution n. 73/2024 dated 17 December 2024. This approval ensures the safeguarding of participants’ rights, dignity, integrity, and well-being. Participants’ anonymity and confidentiality will be strictly maintained during observations and data handling, and any data collected will be used solely for the purpose of evaluating the training program’s effectiveness and its further development.

## 3. Results

The structured methodology for this educational intervention is designed to evaluate its immediate effectiveness, with the following key outcomes. The effectiveness of the training was objectively measured using a specifically developed efficacy scale to quantify participants’ observable behaviors during simulated conflict scenarios. The results, as shown in the table of Tukey’s hinges (Table 3), demonstrate a significant improvement in all assessed skills from the pre-training to the post-training evaluation. For every item on the scale, the median scores increased, indicating a positive shift in overall group performance. The *p*-value for each item was <0.001, confirming that the observed improvements were statistically significant. These results demonstrate enhanced conflict resolution skills, improved communication, and an increased sense of self-efficacy among participants.

## 4. Discussion

This multidisciplinary, occupational medicine-based intervention protocol offers a robust and adaptable framework for addressing the critical issue of workplace conflict and aggression across various high-stress professional environments. Its design, developed by a team of occupational physicians and psychiatrists, reflects a comprehensive understanding of both the psychological and practical dimensions of managing challenging interactions, grounded in an occupational health perspective that prioritizes worker well-being and safety.

### 4.1. Findings and Implications

The results of our study demonstrated a statistically significant increase in conflict management skills. This improvement translates into a number of multifaceted benefits: enhanced communication, a reduction in professional stress and burnout, and an increased sense of safety and self-efficacy in difficult situations. At the organizational level, the effectiveness of the training can lead to a more positive and productive work environment, reduced staff turnover, improved client relations, and ultimately, enhanced service quality. The proactive management of psychosocial risks, as facilitated by this training, contributes directly to a healthier and more sustainable workforce.

### 4.2. Strengths of the Protocol

Several features distinguish this protocol:

Multidisciplinary Expertise: The involvement of occupational physicians and psychiatrists ensures a holistic approach, addressing not only the behavioral aspects of conflict but also the psychological impact on professionals and the broader occupational health implications. This integrated perspective is crucial for effective psychosocial risk management and distinguishes the protocol as being rooted in occupational health principles.

Evidence-Informed Design: The theoretical framework and practical strategies are informed by contemporary research on workplace aggression and de-escalation. Notably, the program’s pedagogical approach is directly aligned with findings from our scoping review [12], which systematically identified and validated effective training methods for managing aggressive behavior in professional settings, particularly the value of active, simulated learning.

Active and Immersive Learning: The emphasis on tailored role-playing sessions provides a safe yet realistic environment for participants to practice and internalize learned skills. This active learning approach is widely recognized as more effective than purely didactic methods for skill acquisition and retention, particularly for complex interpersonal behaviors. The structured observation by external raters adds an objective dimension to this immersive experience.

Focus on Practical Application: The program prioritizes actionable strategies for de-escalation and crisis management, moving beyond abstract concepts to deliver tangible skills that professionals can immediately apply in their daily work.

Preventative Approach: By equipping professionals with effective tools before severe incidents occur, the protocol serves as a proactive strategy for psychosocial risk prevention, contributing significantly to a safer and healthier workplace culture. This aligns with the core mandate of occupational medicine.

Scalability and Adaptability: The clear, modular structure allows for straightforward customization and implementation across various professional contexts, demonstrating its potential for widespread impact.

### 4.3. Limitations and Future Directions

While this protocol provides a robust framework for effective training intervention, it is important to acknowledge certain limitations and outline future research directions. The current protocol primarily focuses on the design of the intervention and its immediate assessment through traditional role-playing. While valuable for gauging skill acquisition, this approach provides a snapshot of acquired competencies rather than long-term behavioral change or sustained impact on real-world incidents. Furthermore, traditional role-playing, by its nature, can sometimes lack the full realism of live interactions, is often time-consuming to prepare, and its efficacy can vary significantly based on individual participants, limiting standardization and the range of usable scenario scripts.

Future research should take the following aims:

- Conduct longitudinal studies to assess the sustained impact of the training on professional well-being, the actual incidence of conflict-related incidents, and employee retention over time. This would provide stronger evidence of the program’s long-term efficacy.
- Integrate comprehensive quantitative and qualitative feedback from participants themselves, beyond just rater observations, to gain a deeper understanding of their subjective experience, perceived skill improvement, and the program’s practical utility.
- Explore and develop advanced technological modalities for the training program. A key future goal is the transfer of the role-playing experience into immersive virtual reality (VR) environments, interfaced with artificial intelligence (AI) to drive avatar behavior. This aims to overcome the inherent artificiality of traditional simulations, providing a more realistic, adaptable, and impactful learning experience for conflict management. This development aligns with our previous research into the potential of VR in occupational stress disorders rehabilitation [20].
- Expand the implementation to larger cohorts across multiple organizations within diverse sectors to further validate the generalizability and scalability of the protocol across various professional settings and to perform comparative analyses.

## 5. Conclusions

The multidisciplinary, occupational medicine-based intervention protocol presented herein offers a valuable and highly adaptable framework for enhancing conflict prevention and crisis management skills in a wide range of high-stress professional environments. By integrating evidence-informed theoretical knowledge with practical, immersive training through tailored role-playing scenarios, it provides a proactive and effective strategy for promoting professional resilience, fostering improved communication, and contributing to safer, more supportive workplaces. This protocol stands as a significant contribution to the field of occupational health, offering a replicable model for organizations committed to mitigating psychosocial risks and supporting the well-being of their workforce, and also laying the groundwork for advanced, technologically enhanced training methodologies in the future.

## Figures and Tables

**Table 1:** Schedule of the project phases.

SCHEDULE OF THE PROJECT	
Months	Project Phases
*1–6*	Literature review to design the program with an evidence-based approach.
*7–8*	Translation of the DABS, adaptation for implementation (adding the eighth item on non-verbal language), and supervision by a sociologist.
*9–10*	University Bioethics Committee approval process.
*11–12*	Protocol presentation to the university and obtaining approval for specific training.
*13*	Selection of the starting department (Veterinary), chosen based on the highest scores in the work-related stress risk assessment and focus group results that identified conflicts as the main problem.
*14*	Program presentation to department staff, collection of sign-ups for role-playing, and booking classrooms for theoretical and practical training.
*15–16*	Execution of the training program for all department staff.
*17–18*	Analysis of the efficacy database to evaluate results.
*19*	Drafting and writing of the scientific article.
**Intervention phases**	
*Role-playing 1*	A brief, approximately 5 min session to assess baseline skills
*Theoretical Training*	2 h of structured theoretical instruction. The content is universally applicable and includes key topics such as the dynamics and phases of aggression, and effective communication strategies for de-escalation
*Role-playing 2*	A second individual 5 min sessions, using new scenarios of similar complexity. It’s designed to assess the practical application of newly acquired skills. After the session, participants receive personalized feedback

**Table 2:** Demographic characteristics of the study population (N = 73).

Job Function	N°	%
Clinical University staff	9	12.3
Non clinical University staff	28	38.4
Technical staff	21	28.8
Administrative staff	15	20.5
Total	73	100.0
**Age**	**N°**	**%**
20–49	26	35.6
50–64	42	57.5
≥65	5	6.8
Total	73	100,0

**Table 3:** Pre- and post-training changes in IMDABS items (Likert Scale) with Tukey’s hinges (N°73).

Tukey’s Hinges	Pre-Training	Post-Training	
IMDABS Items	Median (Q_1_–Q_3_)	Median (Q_1_–Q_3_)	* *p*-Value
*Validation*	2 (1–3)	3 (2–4)	*p* < 0.001
*Reducing emotion*	2 (1–3)	3 (2–4)	*p* < 0.001
*Inquiring about motivations*	2 (1–3)	3 (2–4)	*p* < 0.001
*Providing explanations*	2 (1–3)	3 (2–4)	*p* < 0.001
*Developing solutions*	3 (1–3)	3 (2–4)	*p* < 0.001
*Managing one’s own emotions*	3 (2–3)	4 (3–5)	*p* < 0.001
*Reducing risks*	3 (2–4)	4 (3–4)	*p* < 0.001
*Non-verbal language*	3 (2–3)	4 (2–4)	*p* < 0.001

* Statistical significance *p* ≤ 0.05.

## Data Availability

No new data were created or analyzed in this study protocol. Data sharing is not applicable to this article.
